# Insight into DNA substrate specificity of PARP1-catalysed DNA poly(ADP-ribosyl)ation

**DOI:** 10.1038/s41598-020-60631-0

**Published:** 2020-02-28

**Authors:** Elie Matta, Assel Kiribayeva, Bekbolat Khassenov, Bakhyt T. Matkarimov, Alexander A. Ishchenko

**Affiliations:** 1 Laboratoire «Intégrité du Génome et Cancers» CNRS, UMR9019, Université Paris-Saclay, F-94805 Villejuif, France; 2 Gustave Roussy, Université Paris-Saclay, F-94805 Villejuif, France; 3 National Center for Biotechnology, Nur-Sultan, 010000 Kazakhstan; 4 L.N. Gumilyov Eurasian National University, Nur-Sultan, 010000 Kazakhstan; 5 National Laboratory Astana, Nazarbayev University, Nur-Sultan, 010000 Kazakhstan

**Keywords:** DNA repair enzymes, Transferases, Enzyme mechanisms, DNA-binding proteins

## Abstract

DNA-dependent poly(ADP-ribose) polymerases (PARPs) PARP1, PARP2 and PARP3 act as DNA break sensors signalling DNA damage. Upon detecting DNA damage, these PARPs use nicotine adenine dinucleotide as a substrate to synthesise a monomer or polymer of ADP-ribose (MAR or PAR, respectively) covalently attached to the acceptor residue of target proteins. Recently, it was demonstrated that PARP1–3 proteins can directly ADP-ribosylate DNA breaks by attaching MAR and PAR moieties to terminal phosphates. Nevertheless, little is still known about the mechanisms governing substrate recognition and specificity of PARP1, which accounts for most of cellular PARylation activity. Here, we characterised PARP1-mediated DNA PARylation of DNA duplexes containing various types of breaks at different positions. The 3′-terminal phosphate residue at double-strand DNA break ends served as a major acceptor site for PARP1-catalysed PARylation depending on the orientation and distance between DNA strand breaks in a single DNA molecule. A preference for ADP-ribosylation of DNA molecules containing 3′-terminal phosphate over PARP1 auto-ADP-ribosylation was observed, and a model of DNA modification by PARP1 was proposed. Similar results were obtained with purified recombinant PARP1 and HeLa cell-free extracts. Thus, the biological effects of PARP-mediated ADP-ribosylation may strongly depend on the configuration of complex DNA strand breaks.

## Introduction

One of the earliest DNA damage response events in the cell is the recruitment of DNA-dependent poly(ADP-ribose) polymerases 1, 2 and 3 (PARP1–3) to the sites of DNA strand breaks^1–3^. PARPs 1–3 are catalytically activated through interaction with DNA strand discontinuities and catalyse poly(ADP-ribosyl)ation (PARylation) or mono(ADP-ribosyl)ation (MARylation, in case of PARP3) of nuclear acceptor proteins including auto-ADP-ribosylation using NAD^+^ as the ADP-ribose donor^4–6^. Protein ADP-ribosylation provides a scaffold for the recruitment of other proteins, which also become potential targets for PARP-dependent ADP-ribosylation altering the function of the modified proteins and coordinating the choice of DNA break processing and repair pathways. PARP1 is one of the most abundant nuclear proteins and accounts for ~80–90% of the PARylation activity in the cell induced by DNA damage^7,8^. PARP1 is recruited to damage sites in genomic DNA within a few seconds after laser micro-irradiation^3^ and modulates multiple pathways involved in DNA strand break repair: base excision repair, nucleotide excision repair, homologous recombination and non-homologous end-joining^4,9^. Depletion of PARP1 results in hypersensitivity to ionising radiation, to oxidative stress, and to alkylating agents^10^.

Recently, the previously unknown phenomenon of post-replicative reversible ADP-ribosylation of DNA strand break termini catalysed by mammalian PARP1–3 was uncovered. These PARPs catalyse covalent addition of ADP-ribose units to 5′- and 3′-terminal phosphates and to 2′-OH termini of modified nucleotides at DNA strand breaks, thereby producing a covalent MAR–DNA or PAR–DNA adduct^11–13^. This discovery provides novel molecular insights into PARPs’ functions. Previously, we have partially characterised these activities *in vitro* and obtained the first indirect evidence of the presence of PAR–DNA adducts in human cells after a genotoxic treatment^12^. We have demonstrated that PARP2- and PARP3-catalysed DNA ADP-ribosylation proceeds in a nick/gap-oriented manner and necessitates the presence of at least two DNA strand breaks separated by a distance of 1–2 helix turns.

The protein PARylation activity of PARP1 has been found to be activated by different types of lesions and DNA structures including single- and double-strand DNA breaks (SSBs and DSBs, respectively), DNA crosslinks, stalled replication forks, DNA hairpins, cruciforms, stably unpaired regions and other non-B-conformations of DNA^14^, but the mechanism governing substrate recognition and specificity of PARP1-dependent DNA PARylation is still undetermined. Here we further characterised the mechanism and optimal configuration of DNA structures and breaks for PARP1-catalysed ADP-ribosylation of DNA. We proposed a model of DNA break–oriented binding of PARP1 and demonstrated that PARP1 can catalyse ADP-ribosylation of 3′-phosphorylated DSB termini of the DNA molecules mimicking DSB and SSB breaks even more effectively than auto-PARylation. Possible functional interactions between PARP1-mediated PARylation and formation of 3′-phosphorylated breaks are discussed.

## Results

### Preferential PARylation of 3′-terminal phosphate at a DSB site by PARP1

**Figure 1:**
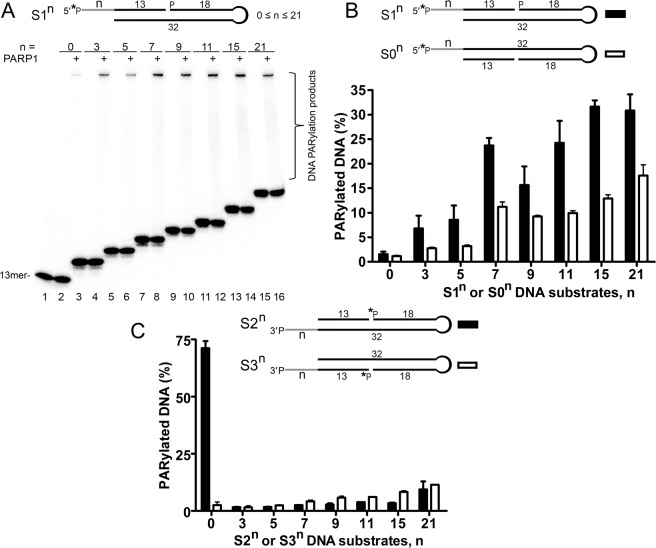
Effects of the type and size of protruding ends in Dbait-based DNA structures containing a 1-nt gap on the PARP1-catalysed formation of PAR–DNA adducts. Twenty-nanomolar PARP1 was incubated with 20 nM 5′-[^32^P]labelled oligonucleotide and 1 mM NAD^+^ for 15 min at 37 °C under standard reaction conditions. (**A**) Denaturing PAGE analysis of PARP1-generated products of PARylation of [^32^P]labelled DNA substrates S1^n^. (**B**) and (**C**) Comparison of DNA PARylation activities of PARP1 towards DNA substrates containing 5′-overhangs (S0^n^ and S1^n^) or 3′-overhangs (S2^n^ and S3^n^), respectively. The data on PARP-catalysed formation of PAR-DNA products are presented as mean ± SD from three independent experiments.

**Figure 2:**
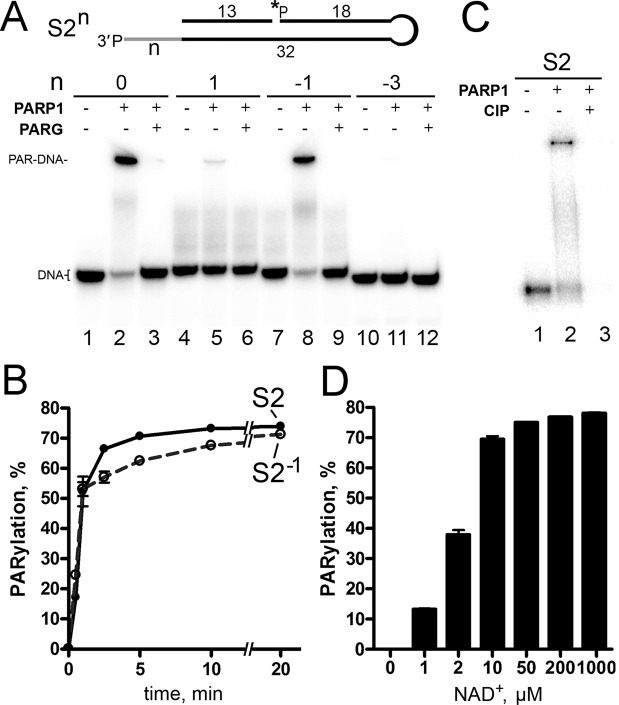
PARP1-catalysed PARylation of S2^n^ DNA structures with a 3′-phosphate terminus at the DBS end. (**A**) [^32^P]labelled DNA substrates S2^n^ (50 nM) were incubated with 40 nM PARP1 for 10 min 37 °C. (**B**) Time dependence of PARP1-driven PARylation of substrates S2 and S2^-1^. DNA substrates (50 nM) were incubated with 20 nM PARP1 for the indicated period under standard reaction conditions. (**C**) CIP-induced dephosphorylation of 5′-[^32^P]labelled PAR-S2 products. After incubation with PARP1, the S2 samples were heated for 10 min at 85 °C, and the resulting [^32^P]labelled DNA PARylation products were further incubated with 10 U of CIP for 30 min at 37 °C. (**D**) The dependence of S2 DNA (40 nM) PARylation by PARP1 (20 nM) on NAD^+^ concentration. The data in panels C and D are presented as mean ± SD from three independent experiments.

Previously, we have demonstrated that PARP1 preferentially ADP-ribosylates 5′-terminal phosphates of single-stranded (ss) oligonucleotides and of 5′-overhangs of a DSB in recessed DNA duplexes^11,12^. Notably, the 2′-hydroxyl group of cordycepin at the 3′ end of a recessed DNA is also targeted by PARP1 for covalent PARylation^11^. Nevertheless, PARP1-mediated DNA ADP-ribosylation of DNA substrates tested until now is still much less effective than PARP2- or PARP3-catalysed PARylation of their preferred DNA substrates^11,12^. In the present study, we further characterised PARP1 DNA substrate specificity and the mechanism of its DNA PARylation activity. It has been demonstrated elsewhere that PARP2- and PARP3-catalysed DNA ADP-ribosylation is strongly dependent on the distance between breaks in DNA substrates^12^. Thus, for optimisation of PARP1 DNA PARylation activity we performed an *in vitro* assay at a saturating concentration of NAD^+^ (1 mM) with the human PARP1 enzyme and various ^32^P-radiolabelled Dbait-based DNA structures (Supplementary Table S1) containing a one-nucleotide (nt) gap for PARP1 activation and 5′- or 3′-terminal phosphates as acceptor groups of various overhangs at a unique DSB end (the opposite DSB terminus ended with a hexaethyleneglycol loop). The reaction products were analysed by denaturing PAGE. As shown in Fig. 1, in case of a 1-nt gap situated 13 nt downstream of the 5′ DSB terminus, effective PARylation of the 5′-terminal phosphate started when 5′-overhangs were ≥7 nt, resulting in a 24–34% yield of PARylated products (S1^n^ DNA substrates, n ≥ 7; Fig. 1A,B). Similar results were obtained in the presence of a physiological non-saturating concentration of NAD^+^ (50 µM) and a 2.5-fold–increased concentration of PARP1 (Supplementary Fig. S1) suggesting that speed of PARP1-catalysed PAR formation does not significantly affect DNA substrate specificity. Notably, HPF1 (histone PARylation factor 1), a PARP1’s interacting partner that is known for modulation of target specificity of PARP1 to serine residues, did not affect PARP1 activity towards the S1^7^ substrate or its profile towards S1^n^ DNA substrates (Supplementary Fig. S2). Substrates S0^n^ mimicking substrates S1^n^ but containing a gap on the opposite strand were less effectively PARylated than S1^n^ were; however, S0^n^ showed a similar profile of the PARylation dependence on the length of 5′ overhangs (Fig. 1B). In contrast to S0^n^ and S1^n^, 3′-phosphorylated protruding termini in DNA substrates S2^n^ and S3^n^ were not effectively PARylated by PARP1 even at n = 21 (Fig. 1C). By contrast, surprisingly, we found that an S2 (S2°) DNA substrate containing 3′-phosphate at the blunt DSB terminus was PARylated very effectively (72% of the product; Fig. 1C). Notably, we did not observe significant modification of 3′-phosphorylated termini when the 1-nt gap was placed on the same, 3′-terminus–containing strand of the duplex (S3^n^ substrates, Fig. 1C). Furthermore, we compared PARP1 activity towards substrates S2, S2^1^, S2^−1^ and S2^−3^ (Fig. 2A) to verify how sensitive PARP1 is towards the position of the 3′-phosphate in S2-based DNA duplexes. The results revealed drastic inhibition of DNA PARylation in case of substrates with a 1-nt 3′-overhang or 3-nt recessed 3′ terminus (S2^1^ and S2^−3^, respectively; Fig. 2A). Only substrate S2^−1^ was as effective as substrate S2 was (Fig. 2A,B), suggesting strict necessity of PARP1 for a 3′-phosphorylated blunt or 1-nt recessed DSB terminus when the gap is positioned on the opposite strand of the DNA duplex 13 nt downstream from the 5′ terminus of DSB. Modification of an unlabelled 3′-terminal phosphate and not of a 5′-[^32^P]labelled terminus in the S2 molecule was confirmed by calf intestinal alkaline phosphatase (CIP) treatment of the reaction products (Fig. 2C). PARP1 kinetic experiments uncovered rapid 3′-phosphate modification with a majority of S2 and S2^−1^ DNA substrates PARylated already after the first minute of the reaction (Fig. 2B) and continued to be effective at low (down to 2 µM) concentrations of NAD^+^ (Fig. 2D).

### An SSB-oriented mechanism of PARP DNA PARylation

**Figure 3:**
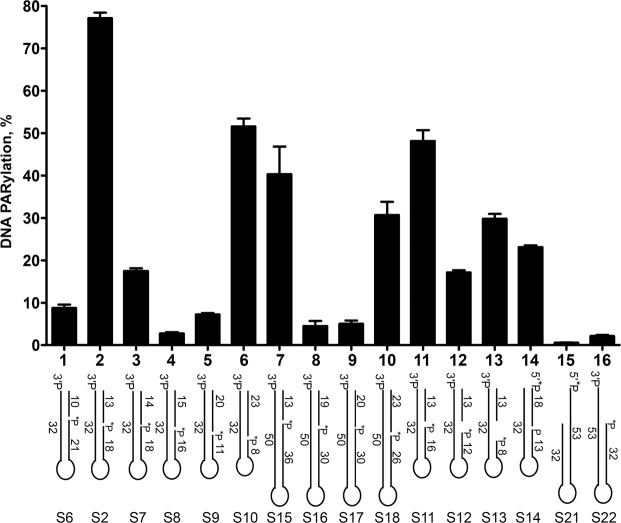
Gap–DSB distance dependence of PARP1-catalysed PARylation of the 3′ phosphate at a DSB end of DNA duplexes. The data on PARP-catalysed formation of PAR-DNA products are presented as mean ± SD from three independent experiments performed under standard reaction conditions.

It has been demonstrated that the accessibility of terminal phosphates of a DSB for PARP2 and PARP3 catalytic sites depends on the distance from a downstream nick^12^. Here, we assessed the dependence of PARP1-catalysed 3′-terminal phosphate PARylation on the distance between a gap and a blunt DSB end in Dbait-based DNA structures of different lengths. As presented in Fig. 3, among substrates tested in columns 1–10, only DNA substrates containing a gap at a 13- or 23-nt distance from DSB termini (substrates S2 and S15 or S10 and S18, respectively) were PARylated effectively. Notably, the extent of DNA-PARylation was very sensitive to the distance between the DSB and the 3′ end of the SSB because attachment of a single nucleotide to the S7 substrate resulted in a strong reduction of DNA PARylation in comparison to the S2 substrate. Taking into account that the 10-nt difference in the distance represents one turn of the DNA helix, these data suggest that the position of the acceptor phosphate relative to SSB in the DNA helix plays a discriminating role for PARP1-dependent modification, as observed previously in the case of PARP2 and PARP3 enzymes. This conclusion was confirmed by significant PARylation of a 5′-terminal phosphate observed at a blunt-ended DSB in the S14 substrate (Fig. 3, column 14) but not in the S1° substrate (Fig. 1), which feature a half-DNA helix difference in the positions of their gaps (18 and 13 nt downstream from the DSB end, respectively). The increased size of gaps in substrates S11, S12, and S13 (3, 7 and 11 nt, respectively) resulted in a significantly lower DNA PARylation yield (17–48%) as compared to the S2 substrate (77%) containing a 1-nt gap (Fig. 3, columns 11–13 versus 2). Modification of an unlabelled 3′-terminal phosphate at DSB end in substrates S10-13 was confirmed by CIP treatment of the reaction products (Supplementary Fig. S3). These results indicated that PARP1-dependent DNA PARylation of the 3′-terminal phosphate at the DSB terminus is not restricted to DNA duplexes with short gaps although the 1-nt gap apparently better coordinates PARP1 binding and activation for subsequent PARylation of such DNA substrates.

### The monomeric mode of PARP1 binding to Dbait-based molecules prone to PARylation

**Figure 4:**
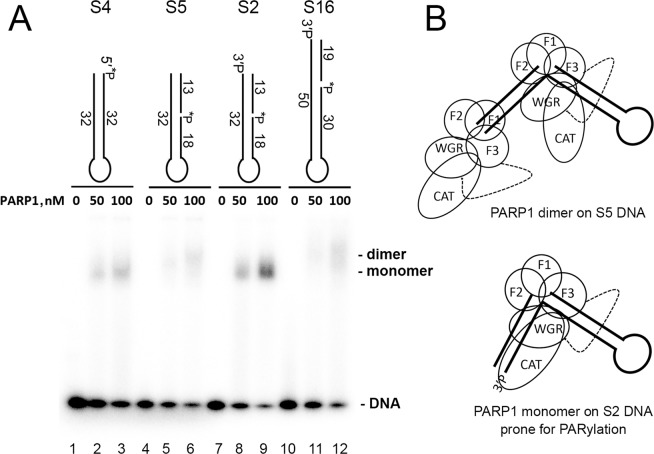
The monomeric mode of PARP1 binding to DNA molecules prone to PARylation. (**A**) The EMSA. Each of 20 nM DNA duplexes was incubated with 0, 50 or 100 nM PARP1 in a buffer consisting of 20 mM Tris-HCl pH 7.6, 50 mM KCl and 1 mM DTT for 10 min at room temperature. The DNA–protein complexes were analysed by electrophoresis in a 4–12% Tris-Glycine polyacrylamide gel (Novex) under non-denaturing conditions at 4 °C after addition of 10% glycerol. (**B**) The putative model of PARP1 complexes with DNA substrates prone (S2) or not prone (S5) to DNA break PARylation.

DNA molecules prone to ADP-ribosylation contain at least two proximal breaks for PARP activation and terminal phosphate modification. It has been suggested that PARP affinity for the modification site should be relatively low to prevent the binding of a second PARP molecule, which can sterically protect this site from modification^11,12^. Taking into account that both DSB blunt ends and an SSB have high affinities for PARP1^15,16^, the effective PARylation of DNA substrates S2 and S14 raises a question: Does PARP1 bind to blunt-ended DSB termini of such substrates? A gel electromobility shift assay (EMSA) of 5′-[^32^P]labelled DNA substrates S2, S4, S5 and S16 in the presence of various PARP1 concentrations showed that PARP1 complexes with S2 and control ungapped S4 migrated as single bands and had similar electromobility (Fig. 4A, lanes 8, 9 and 2, 3, respectively). On the contrary, PARP1 complexes with substrate S5 (the same as S2 but the 3′-phosphate is absent) and S16 (contains the 3′-phosphate but at a position not prone to PARylation) migrated notably more slowly as diffuse doublets (Fig. 4A, lanes 6 and 12, respectively) indicating a binding of an additional PARP1 molecule. Previously, formation of 1:1 or 2:1 PARP1–DNA complexes on EMSA gels has been demonstrated with 53-bp blunt-ended DNA duplexes^17^. These data are suggestive of the monomeric mode of PARP1 binding to the S2 DNA substrate (Fig. 4B). Intramolecular accommodation of the phosphorylated DSB terminus of the S2 substrate in the catalytic site of PARP1 bound to the gap on the same DNA molecule, in our opinion, could explain the observed “hiding” of the DSB terminus of the S2 substrate from the binding of an additional PARP1 molecule and consequently its effective PARylation.

### A 3′-phosphorylated DSB terminus is a major acceptor site of PARylation as compared to PARP1 auto-ADP-ribosylation

**Figure 5:**
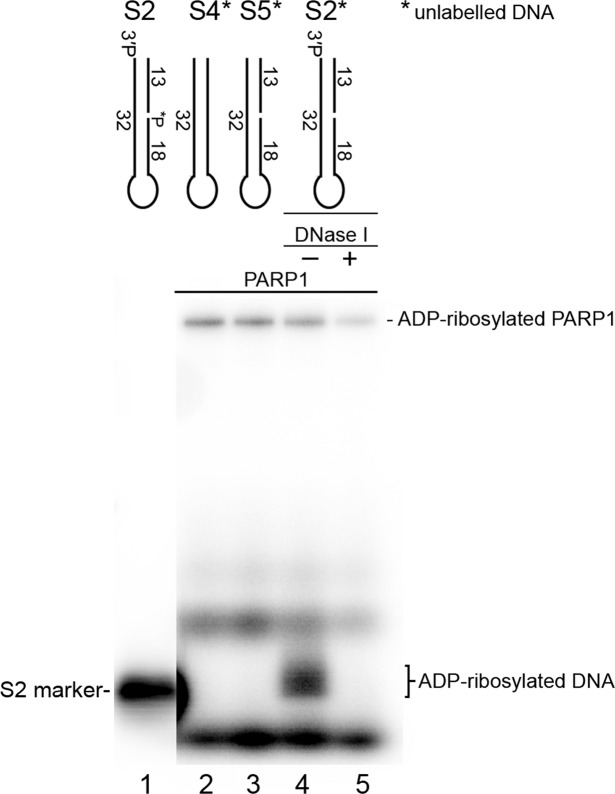
Comparison of the efficiency of PARP1-catalysed auto- and DNA ADP-ribosylation. The denaturing SDS-PAGE analysis of the products of PARP1 incubation with cold oligonucleotide duplexes in the presence of [adenylate-^32^P]NAD^+^. For details see Materials and Methods.

Previously, we demonstrated preferential DNA break modifications by enzymes PARP2 (~5-fold) and PARP3 (~50-fold) as compared to their auto-ADP-ribosylation if the DNA substrates are prone to ADP-ribosylation^12^. Nevertheless, PARP1 modification of DNA breaks in our *in vitro* assays has always been at least 10-fold less effective than simultaneous auto-ADP-ribosylation^11,12^. Here, we incubated unlabelled DNA substrates S2, S4 and S5 with PARP1 in the presence of a low concentration of [adenylate-^32^P]NAD^+^ and separated both types of ADP-ribosylation products by SDS-PAGE (Fig. 5). The results indicated that PARP1 was efficiently auto-ADP-ribosylated in the presence of any DNA substrates tested, but a DNA ADP-ribosylation product was observed only in case of the S2 construct containing the 3′-phosphate at the DSB terminus. The yield of S2 DNA ADP-ribosylation was ~5-fold higher as compared to PARP1 auto-ADP-ribosylation in the same reaction mixture (Fig. 5, lane 4), suggesting that PARP1-catalysed DNA ADP-ribosylation can be even more effective than its auto-ADP-ribosylation in case of an optimal configuration of proximal DNA strand breaks.

### 3′-Phosphorylated DNA substrates are PARylated in human cell-free extracts

**Figure 6:**
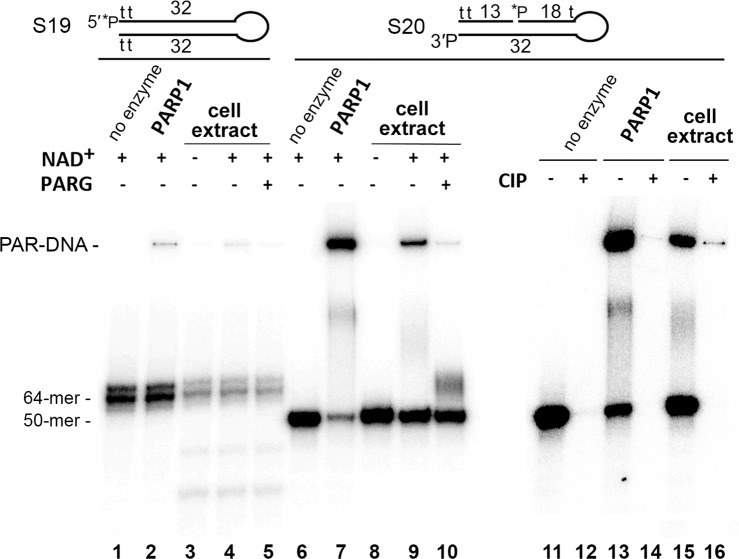
Formation of PAR–3′ phosphate–DNA adducts in nuclear extracts from HeLa PARG^KD^ cells. Fifty-nanomolar [^32^P]labelled S19 or S20 Dbait-based molecules were incubated with 2.5 µg/µl HeLa extracts or 40 nM PARP1 in the presence of 67 mM KCl, 10 mM HEPES-KOH pH 8.0 and 500 µM NAD^+^ for 20 min at 37 °C. The reactions were stopped by heating the samples for 10 min at 80 °C, and the resulting DNA PARylation products were next incubated with 20 pg/µl PARG (lanes 5 and 10) or after phenol-chloroform extraction with 10 U of CIP for 30 min at 37 °C (lanes 12, 14 and 16). The reaction products were analysed by denaturing PAGE.

To test the possibility of the 3′-terminal phosphate ADP-ribosylation at a DSB site in extracts of human cells, we used DNA substrates S19 and S20 mimicking constructs S4 and S2, respectively, but containing several internucleotide thiophosphate linkages for protection against nuclease degradation (Supplementary Table S1). As depicted in Fig. 6, the 5′-[^32^P]labelled S19 control substrate without a gap and 3′-terminal phosphate group was not effectively PARylated in HeLa PARG^KD^ cell-free extracts and partially degraded (lanes 3–5). In contrast, the 5′-[^32^P]labelled S20 substrate with a 1-nt gap, 3′-terminal phosphate, and an additional thiophosphate linkage at the site of the hexaethyleneglycol loop was effectively PARylated and not degraded in the condition tested (lanes 9, 10 and 15). PARylation of the 3′- but not 5′-[^32^P]labelled phosphate in the S20 substrate was confirmed by additional CIP treatment that completely removed the unprotected [^32^P]labelled phosphate of PARylated products (lanes 14 and 16). These results are in agreement with the data obtained in our previous work showing effective PARP1-dependent PARylation of a 5′-terminal phosphate in a 5′-overhang of an S1^7^-like DNA molecule in human cell-free extracts^12^. Altogether, these results suggest that the DNA PARylation activity in the HeLa cell-free extracts can be efficient towards both 3′- and 5′-terminal phosphates depending on the structure of DNA breaks.

## Discussion

PARP1 is an abundant, ubiquitously expressed nuclear protein that has long been regarded as a central DNA damage–responsive factor in mammalian cells that is required for the maintenance of genome integrity^3,18^. It is generally accepted that PARP-dependent PARylation of chromatin results in chromatin remodelling facilitating the assembly of repair complexes at SSBs and DSBs^4,19^. PARylation and other PARP1-mediated events are critically involved not only in DNA damage repair but also in a wide array of other biological processes, including replication, epigenetic regulation, transcription, apoptosis, inflammation, RNA metabolism, autophagy and proteasomal activation^20–23^. Other DNA-dependent proteins, PARP2 and PARP3, have their specific and partially redundant functions relative to PARP1, and all three enzymes often act synergistically in response to genotoxic stress^2,10^. The number of known PARP functions in the cell continues to grow. For example, recent work from Caldecott’s laboratory indicates that in unperturbed cells, PARP1 is a sensor of unligated Okazaki fragments during DNA replication and facilitates their repair^24^. Other functions still need to be clarified, including the roles of reversible ADP-ribosylation of DNA catalysed by PARP1–3 and MARylation of 5′-phosphorylated termini of RNA molecules by PARP10, PARP11, PARP15 and TRPT1 recently demonstrated in *in vitro* studies^11–13,25^.

Despite prior insights into PARP1 DNA PARylation activity, there remain key questions regarding the regulation of PARP1 activity, including the mechanism and specific requirements for its unusual substrate specificity towards DNA breaks. PARP1 activity towards previously tested DNA substrates is relatively slow and not very effective as compared to the DNA ADP-ribosylation activity of enzymes PARP2 and PARP3^11,12^, thus casting a reasonable doubt on the biological relevance of this PARP1 activity. Here, we show that PARP1 very effectively PARylates a 3′-terminal phosphate at a DSB site of gapped DNA duplexes thereby producing more than 50% of PARylated DNA products already after 1 min of incubation at relatively low (20 nM) enzyme concentrations (Fig. 2B). This activity is effective in a wide range (2–1000 µM) of NAD^+^ concentrations (Fig. 2D). Taking into account that the NAD^+^ concentrations in the nucleus and cytoplasm are estimated to be ~100 μM^26^, these results support the potency of PARP1-dependent PARylation of specific DNA breaks in the cell. This notion is also supported by the PARylation of 3′-phosphorylated DNA breaks in cell-free extracts (Fig. 6) and by the results of the parallel measurement of PARP1-mediated auto- and DNA PARylation, revealing even more efficient modification of the 3′-phosphate of the S2 DNA substrate prone to PARylation as compared to simultaneous PARP1 auto-PARylation (Fig. 5). A similar observation has been made previously regarding PARP2-mediated and PARP3-mediated ADP-ribosylation of a 5′-terminal phosphate at a DSB site of nicked DNA duplexes^12^, suggesting that all three DNA-dependent PARPs can preferentially target proximal DNA breaks.

**Figure 7:**
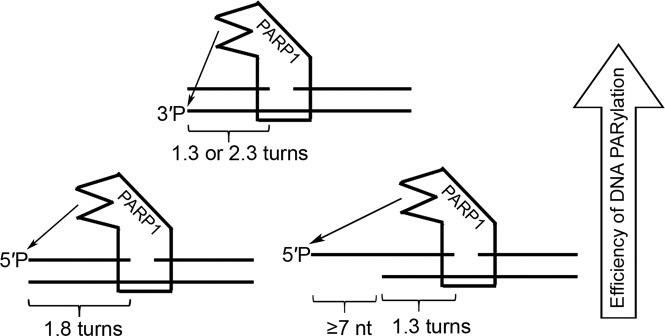
Schematic representation of the putative model of DNA modification by PARP1 activated on a 1-nt gap.

PARP1 is a modular protein and has six distinct folded domains, where three N-terminal zinc finger domains and a tryptophan-glycine-arginine (WGR) domain have been reported to be essential for DNA break binding and DNA-dependent activation of the C-terminal catalytic (CAT) domain^27–30^. Interdomain contacts play a primary role in the allosteric mechanism of catalytic activation of all three DNA-dependent PARPs via local destabilisation of the auto-inhibitory helical subdomain of CAT^31^. In contrast to PARP1, PARP2 and PARP3 do not have zinc finger domains and PARPs 1–3 are differently activated by a variety of damaged DNA structures^32–34^. Notably, PARP2 and PARP3 are preferentially activated by an SSB harbouring a 5′-terminal phosphate, in contract to PARP1, which is activated regardless of the phosphorylation status of the DNA ends^34^. Previously, we proposed a mechanistic model where PARP3-catalysed and PARP2-catalysed DNA ADP-ribosylation depends on the orientations and distances between DNA strand breaks in a single DNA molecule^12^. Accordingly, PARP3 and PARP2 ADP-ribosylate the 5′ DSB terminus of the same nicked strand if these breaks are separated by a distance of one or two turns of the DNA helix and less effectively ADP-ribosylate the 3′-DSB terminus of opposite strands if the breaks are separated by a distance of 1.5 helix turns^12^. The present study shows that PARP1 has different DNA substrate requirements for PARylation of terminal phosphates (1.3 or 2.3 and 1.8 helix turns for 3′- and 5′-DSB blunt termini, respectively) but shows similar dependence on DNA helicity and on the orientations of strand breaks (Figs. 1 and 3). Taken together, these findings suggest that effective DNA ADP-ribosylation depends on an interplay between the activation of a DNA-bound PARPs and accessibility of the DNA acceptor group for their CAT domain. According to these results, we propose a model of PARP1-mediated DNA ADP-ribosylation (Fig. 7) where the binding of PARP1 to an SSB (1-nt gap) activates its catalytic domain, which in turn starts to ADP-ribosylate all sterically accessible acceptor groups in the same DNA–enzyme complex. This model is supported by the observed monomeric mode of PARP1 binding to the S2 DNA substrate prone to effective PARylation despite the presence of two breaks on the same DNA molecule (Fig. 4). Structural studies conducted in Pascal’s and Neuhaus’s laboratories have revealed that PARP1 binds SSBs with directional selectivity, where zinc fingers 1 and 2 bind to 5′ and 3′ stems, respectively, and the distance between a DNA break (DSB or SSB)-binding site and the catalytic site in PARP1–DNA complexes is ~45 Å, which corresponds to ~1.3 turns (≈13 bp) of a B-DNA helix^27,29^. This observation may explain the strong preference of PARP1 for the S2 substrate, in which the distance between a protein-binding SSB and the PAR-accepting DSB termini is 13 bp. Nevertheless, it should be stressed that other DNA substrates with a greater distance between two strand break sites can still be modified by PARP1. We can hypothesise that the efficient ADP-ribosylation of S2, S10, S14 and other DNA substrates is due to (*i*) the position of their acceptor phosphates, which is on the same side of the DNA helix exposed to the active site of the PARP1 CAT domain; (*ii*) the highly dynamic nature of the multi-domain proteins and (iii) the flexibility of ss overhangs in substrates S0^n^ and S1^n^ (with n ≥ 7), which might enable 5′ termini to reach the CAT site. The latter notion is supported by effective ADP-ribosylation of 5′ overhangs in S1^n^ duplexes (with n ≥ 3 nt) – but not that of the blunt S1^0^ duplex – catalysed by PARP2 or PARP3 (Supplementary Fig. S4) relaxing the necessity of a 10 or 20 bp distance between a blunt DSB and an SSB for effective ADP-ribosylation of a 5′ DSB terminus^12^. The absence of PARylation of the S21 substrate (Fig. 3), which mimics S1^21^ but lacks a gap, rules out that PARP1 is activated on ds-ssDNA transitions at 5′ overhangs when it PARylates S1^21^ and other S1^n^ structures. The absence of DNA PARylation of substrates S2^n^ and S3^n^ with 3′ overhangs (Fig. 1) suggests that other structural elements of the acceptor DNA terminus are required for accommodation of the terminal phosphate residue in the active site of the PARP1 CAT domain. Given the strong flexibility of both PARP1 and SSB-containing DNA polymers, it is tempting to speculate that with DNA molecules prone to PARylation, PARP1 will preferentially form structurally different complexes stabilised by interactions with both proximal breaks including the CAT domain interaction with an upstream phosphorylated terminus. Further studies are needed to test this hypothesis.

According to the evidence collected so far, DNA ADP-ribosylation activity of PARPs strongly depends on the type and position of DNA breaks. Proximal DNA breaks can be generated directly by genotoxic agents or during processing of the initial DNA damage by DNA repair and DNA replication machineries. In this study, we demonstrate that PARP1 preferentially PARylates a 3′-phosphate of DSB sites in proximity to an SSB. It should be noted that 3′-phosphate termini can be generated endogenously as an intermediate of the action of bi-functional DNA glycosylases or as a product of a tyrosyl-DNA phosphodiesterase 1 (TDP1) reaction, which can remove a variety of 3′ adducts from an SSB and DSB during DNA repair and leave a 3′-terminal phosphate^35,36^. It has been reported that PARP1 plays a critical part in TDP1-mediated repair of trapped topoisomerase I (TOP1) cleavage complexes^37^. PARP1 directly binds to the N-terminal domain of TDP1 and PARylates TDP1 without blocking its catalytic activity. Multiple studies show that PARP inhibitors are sensitise cells to TOP1 poisoning by camptothecin^38–40^. Moreover, genetic evidence indicates that PARP1 and TDP1 are epistatic for the repair of TOP1–induced DNA damage^37^. We suggest that the PARP1-dependent PARylation of 3′-phosphorylated DNA breaks observed here may further enhance the functional interactions between the PARP1 and TDP1. Additional studies are warranted to elucidate how PARP1-dependent DNA PARylation can perform its specific function in a cellular response to DNA damage.

## Materials and Methods

### Proteins, chemicals and reagents

Proteinase K from *Tritirachium album* and deoxyribonuclease I from bovine pancreas (DNase I) were purchased from Sigma–Aldrich (France), whereas CIP and TdT (terminal deoxynucleotidyl transferase) from New England Biolabs France (Evry, France). Human poly(ADP-ribose) polymerase 1 (PARP1; EC 2.4.2.30) and bovine PARG were bought from Trevigen (Gaithersburg, MD, USA).

### Oligonucleotides and Dbait molecules

Sequences of the oligonucleotides and their duplexes used in this work are shown in Supplementary Table S1. Regular oligonucleotides, oligonucleotides with thiophosphates as well as Dbait molecules containing a hexaethyleneglycol linker [(CH_2_-CH_2_-O)_6_] tethering two complementary DNA strands were acquired from Eurogentec (Seraing, Belgium). Prior to enzymatic assays, the oligonucleotides were labelled at the 5′-OH end using T4 polynucleotide kinase (Thermo Scientific) in the presence of [γ-^32^P]ATP (3000 Ci·mmol^−1^, PerkinElmer) as described previously^11^. Cold ATP at 0.1 mM was added to phosphorylate the remaining non-labelled oligonucleotides. After the labelling reactions, the radioactively labelled oligonucleotides were desalted on a Sephadex G-25 column, equilibrated with water and then annealed with a corresponding complementary strand for 3 min at 65 °C in the following buffer: 20 mM HEPES-KOH (pH 7.6) and 50 mM KCl.

### DNA ADP-ribosylation assay

PARP-dependent DNA ADP-ribosylation activity was measured as described previously^11^. Briefly, one of 20 nM [^32^P]labelled oligonucleotide duplexes was combined with 20 nM PARP1 in the presence of 1 mM NAD^+^ in ADPR buffer (20 mM HEPES-KOH pH 7.6, 50 mM KCl, 2 mM MgCl_2_, 1 mM DTT and 100 µg/ml BSA). The mixture was incubated for 30 min at 37 °C, unless stated otherwise. After the reaction, the samples were incubated with 50 ng*/*µl proteinase K and 0.15% of SDS for 30 min at 50 °C followed by the addition of 4 M urea and incubation for 10 s at 95 °C. The reaction products were analysed by electrophoresis in denaturing 20% (w*/*v) polyacrylamide gels (PAGE; 7 M Urea, 0.5× TBE, 42 °C). A Fuji FLA-3000 Phosphor Screen was exposed to the gels and was then scanned with Typhoon FLA-9500, and the image was analysed in the Image Gauge 4.0 software.

### Simultaneous evaluation of the efficiency of PARP1-catalysed auto- and DNA ADP-ribosylation

This assay was carried out as described previously^12^ with minor modifications. 320 nM PARP1 was added to cold 1 µM oligonucleotide duplex and incubated in ADPR buffer but without BSA in the presence of 0.5 µM [adenylate-^32^P]NAD^+^ (800 Ci·mmol^−1^, PerkinElmer) for 30 min at 37 °C. The reaction products were treated with 10.5 U of DNase I for 30 min at 37 °C in the presence of 0.5 mM CaCl_2_. After heating for 1 min at 95 °C in 1× LDS sample buffer (Invitrogen), the products of the reaction were separated by SDS-PAGE on precast 10% gels (Invitrogen).

### The cell line, culture conditions, and preparation of nuclear extracts

Stable PARG knockdown (shPARG/PARG^KD^) and control (shCTL/BD650) HeLa cell lines have been described elsewhere^41^. The cells were grown in DMEM (Dulbecco’s modified Eagle’s medium) supplemented with penicillin/streptomycin (Gibco, Gaithersburg, USA) and 10% of foetal bovine serum in a humidified atmosphere containing 5% of CO_2_. After harvesting, the cells were washed twice in cold phosphate-buffered saline (PBS). All the procedures were conducted at 4 °C. The cell pellets were resuspended in 3 volumes (w/v) of cytoplasmic extract buffer (10 mM HEPES-KOH pH 7.6, 10 mM KCl, 0.1 mM EDTA, 0.15 mM spermine, 0.7 mM spermidine, 1× cOmplete protease inhibitors EDTA-free [Roche] and 1 mM DTT); 0.1% of NP-40 was added immediately after cell resuspension. The cells were allowed to swell on ice for 5 min. Nuclei were collected by centrifugation (500 × *g*, 5 min), then resuspended in 1 volume of nuclear extract buffer (20 mM HEPES-KOH pH 7.6, 0.4 M NaCl, 1 mM EDTA, 25% of glycerol, 1× cOmplete protease inhibitors EDTA-free [Roche] and 1 mM DTT). After 10-min incubation on ice, the samples were centrifuged at 13 000 × *g* for 5 min. The nuclear extracts (supernatants) were stored at −20 °C if not used immediately.

## Supplementary information

Supplementary Information.

## Data Availability

The raw and processed data are available from the corresponding author on reasonable request.
