# Supplementary material for: Insight into DNA substrate specificity of PARP1-catalysed DNA poly(ADP-ribosyl)ation
Source: Sci Rep. 2020 Feb 28;10:3699. doi: 10.1038/s41598-020-60631-0 (PMC7048826; doi:10.1038/s41598-020-60631-0)
Supplement: Supplementary file 1 — Supplementary Information. [file 41598_2020_60631_MOESM1_ESM.pdf]

Supplementary Table and Figures

Insight into DNA substrate specificity of PARP1 catalyzed DNA poly(ADP-ribosyl)ation

Elie Matta[1,2,†], Assel Kiribayeva[3,4,†], Bekbolat Khassenov[3], Bakhyt T. Matkarimov[5], Alexander A. Ishchenko[1,2]

[1]Laboratoire «Intégrité du Génome et Cancers» CNRS, UMR9019, Université Paris-Saclay, F-94805 Villejuif, France
[2]Gustave Roussy, Université Paris-Saclay, F-94805 Villejuif, France
[3]National Center for Biotechnology, Nur-Sultan, 010000, Kazakhstan.
[4]L.N. Gumilyov Eurasian National University, Nur-Sultan, 010000, Kazakhstan.
[5]National Laboratory Astana, Nazarbayev University, Nur-Sultan, 010000, Kazakhstan.

Table S1. Sequences of the oligonucleotides and their duplexes used in this study.

Name	Oligonucleotides sequences and structures
$S0^n$	5′ 32P-TCATAGGCTTAGTCGTCATTCGCTGTGCCCTCAACCGAATTCACAAGCCTAGA (n)<br>3′ CGACACGGGAGTT GCTTAAGTGTTCGGATCT HEG Linker
$S1^n$	5′ 32P-TCATAGGCTTAGTCGTCATTCGCTGTGCCCTCAA P-CGAATTCACAAGCCTAGA (n)<br>3′ CGACACGGGAGTTGGCTTAAGTGTTCGGATCT HEG Linker
$S2^n$	5′ GCTGTGCCCTCAA 32P-CGAATTCACAAGCCTAGA<br>3′ P-CTTACTGCTGATTCGGATACTCGACACGGGAGTTGGCTTAAGTGTTCGGATCT (n) HEG Linker
S2 ($S2^0$)	5′ GCTGTGCCCTCAA 32P-CGAATTCACAAGCCTAGA<br>3′ P-CGACACGGGAGTTGGCTTAAGTGTTCGGATCT HEG Linker
$S2^{-1}$	5′ GCTGTGCCCTCAA 32P-CGAATTCACAAGCCTAGA<br>3′ P-GACACGGGAGTTGGCTTAAGTGTTCGGATCT HEG Linker
$S2^{-3}$	5′ GCTGTGCCCTCAA 32P-CGAATTCACAAGCCTAGA<br>3′ P-CACGGGAGTTGGCTTAAGTGTTCGGATCT HEG Linker
$S3^n$	5′ GCTGTGCCCTCAACCGAATTCACAAGCCTAGA<br>3′ P-CTTACTGCTGATTCGGATACTCGACACGGGAGTT-32P GCTTAAGTGTTCGGATCT (n) HEG Linker
S4	5′ 32P-GCTGTGCCCTCAACCGAATTCACAAGCCTAGA<br>3′ CGACACGGGAGTTGGCTTAAGTGTTCGGATCT HEG Linker
S5	5′ GCTGTGCCCTCAA 32P-CGAATTCACAAGCCTAGA<br>3′ CGACACGGGAGTTGGCTTAAGTGTTCGGATCT HEG Linker
S6	5′ GCTGTGCCCT 32P-AAGCGAATTCACAAGCCTAGA<br>3′ P-CGACACGGGAGTTGGCTTAAGTGTTCGGATCT HEG Linker
S7	5′ GCTGTGCCCTCAAC 32P-CGAATTCACAAGCCTAGA<br>3′ P-CGACACGGGAGTTG-GCTTAAGTGTTCGGATCT HEG Linker
S8	5′ GCTGTGCCCTCAACC 32P-AATTCACAAGCCTAGA<br>3′ P-CGACACGGGAGTTGGCTTAAGTGTTCGGATCT HEG Linker
S9	5′ GCTGTGCCCTCAACCGAATT 32P-ACAAGCCTAGA<br>3′ P-CGACACGGGAGTTGGCTTAAGTGTTCGGATCT HEG Linker
S10	5′ GCTGTGCCCTCAACCGAATTCAC 32P-AGCCTAGA<br>3′ P-CGACACGGGAGTTGGCTTAAGTGTTCGGATCT HEG Linker
S11	5′ GCTGTGCCCTCAA 32P-AATTCACAAGCCTAGA<br>3′ P-CGACACGGGAGTTGGCTTAAGTGTTCGGATCT HEG Linker
S12	5′ GCTGTGCCCTCAA 32P-CACAAGCCTAGA<br>3′ P-CGACACGGGAGTTGGCTTAAGTGTTCGGATCT HEG Linker
S13	5′ GCTGTGCCCTCAA 32P-AGCCTAGA<br>3′ P-CGACACGGGAGTTGGCTTAAGTGTTCGGATCT HEG Linker
S14	5′ 32P-GCTGTGCCCTCAACCGAA P-TCACAAGCCTAGA<br>3′ CGACACGGGAGTTGGCTTAAGTGTTCGGATCT HEG Linker
S15	5′ GCTGTGCCCTCAA 32P-CGAATTCACAAGCCTAGAGTTACATTAGCAGATACG<br>3′ P-CGACACGGGAGTTGGCTTAAGTGTTCGGATCTCAATGTAATCGTCTATGC HEG Linker
S16	5′ GCTGTGCCCTCAACCGAAT 32P-CACAAGCCTAGAGTTACATTAGCAGATACG<br>3′ P-CGACACGGGAGTTGGCTTAAGTGTTCGGATCTCAATGTAATCGTCTATGC HEG Linker
S17	5′ GCTGTGCCCTCAACCGAATT 32P-CACAAGCCTAGAGTTACATTAGCAGATACG<br>3′ P-CGACACGGGAGTTGGCTTAA-GTGTTCGGATCTCAATGTAATCGTCTATGC HEG Linker
S18	5′ GCTGTGCCCTCAACCGAATTCAC 32P-AGCCTAGAGTTACATTAGCAGATACG<br>3′ P-CGACACGGGAGTTGGCTTAAGTGTTCGGATCTCAATGTAATCGTCTATGC HEG Linker
S19	5′ 32P-GCTGTGCCCTCAACCGAATTCACAAGCCTAGA (t t)<br>3′ CGACACGGGAGTTGGCTTAAGTGTTCGGATCT (t t) HEG Linker
S20	5′ GCTGTGCCCTCAA 32P-CGAATTCACAAGCCTAGA (tt, t)<br>3′ P-CGACACGGGAGTTGGCTTAAGTGTTCGGATCT HEG Linker
S21	5′ 32P-TCATAGGCTTAGTCGTCATTCGCTGTGCCCTCAACCGAATTCACAAGCCTAGA<br>3′ CGACACGGGAGTTGGCTTAAGTGTTCGGATCT HEG Linker
S22	5′ 32P-GCTGTGCCCTCAACCGAATTCACAAGCCTAGA<br>3′ P-CTTACTGCTGATTCGGATACTCGACACGGGAGTTGGCTTAAGTGTTCGGATCT HEG Linker

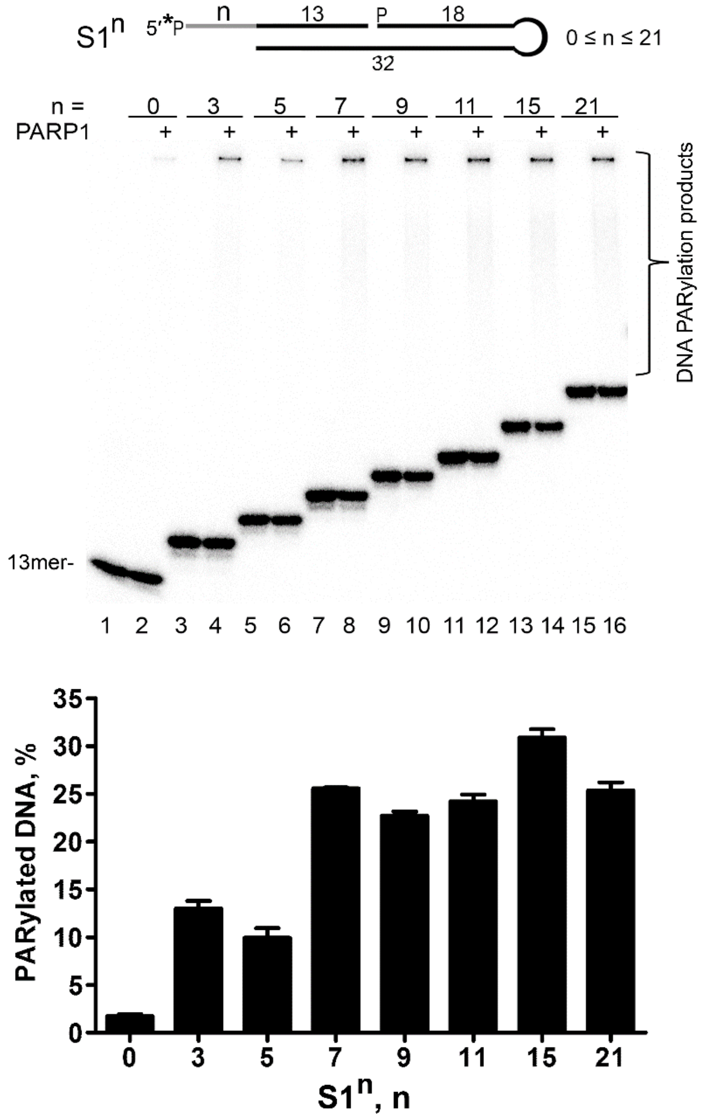

Figure S1. ADP-ribosylation of $S1^n$ DNA duplexes containing 5′-otherhangs by PARP1 at a non-saturating concentration of NAD^+. $[^{32}P]$labelled $S1^n$ DNA duplexes (20 nM) were combined with 50 nM PARP1 in the presence of 50 µM NAD^+ for 15 min at 37°C under standard reaction conditions. The data on PARP-catalysed formation of DNA ADP-ribosylation products are presented as mean ± SD from three independent experiments.

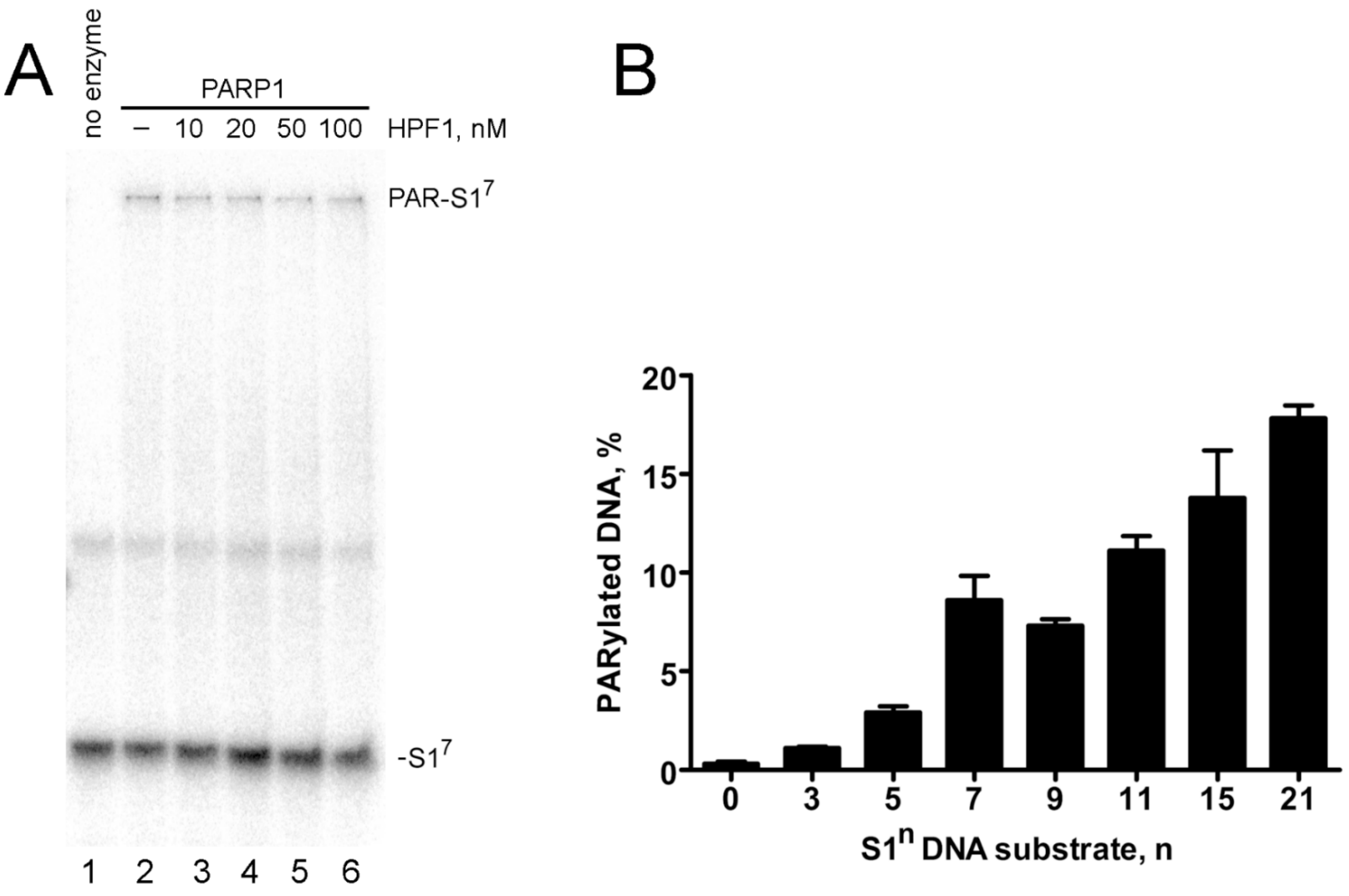

Figure S2. PARP1 DNA PARylation activity in presence of HPF1. (**A**) The 5′-[^{32}P]labelled S1^7 DNA duplex (20 nM) was incubated with PARP1 (10 nM) and varying concentrations of HPF1 under standard reaction conditions. The products of the reaction were separated using denaturing PAGE. (**B**) Comparison of DNA PARylation activities of PARP1 (10 nM) toward S1^n DNA substrates (20 nM) in presence of 100 nM HPF1 under standard reaction conditions. The purified human HPF1 protein was kindly provided by Dr Ivan Ahel (University of Oxford, U.K.). The error bars represent the standard deviation (*n* = 3).

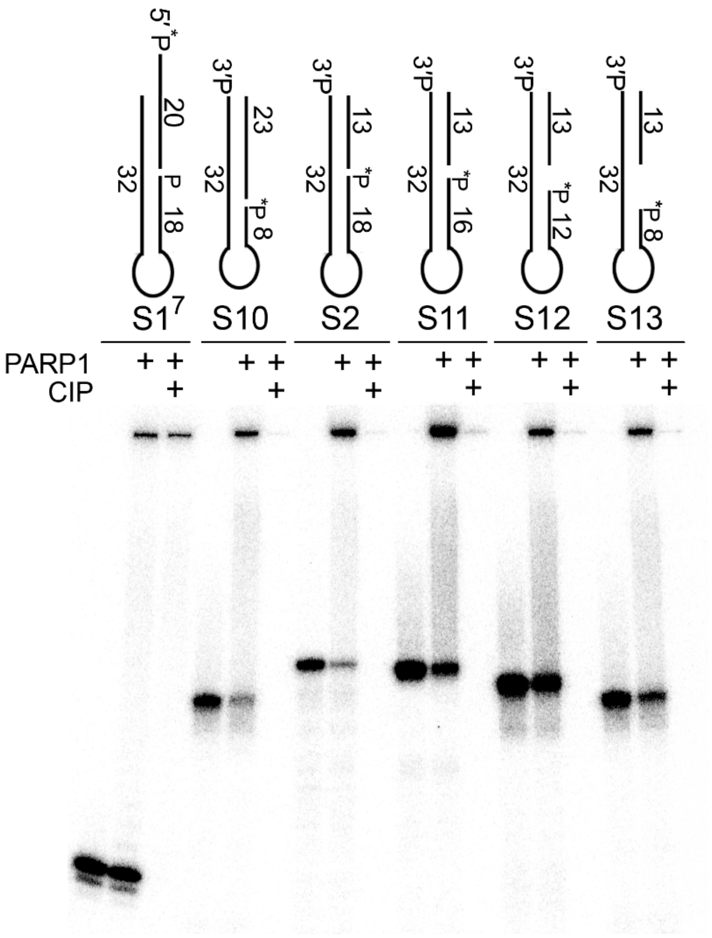

Figure S3. Validation of PARP1-dependent PARylation of 3′-phosphorylated DSB termini of DNA substrates S2 and S10-13 by CIP-induced dephosphorylation. After incubation with PARP1, the 5′-[^{32}P]labelled DNA samples were heated for 10 min at 85°C, and the resulting [^{32}P]labelled DNA PARylation products were further incubated with 10 U of CIP for 30 min at 37°C.

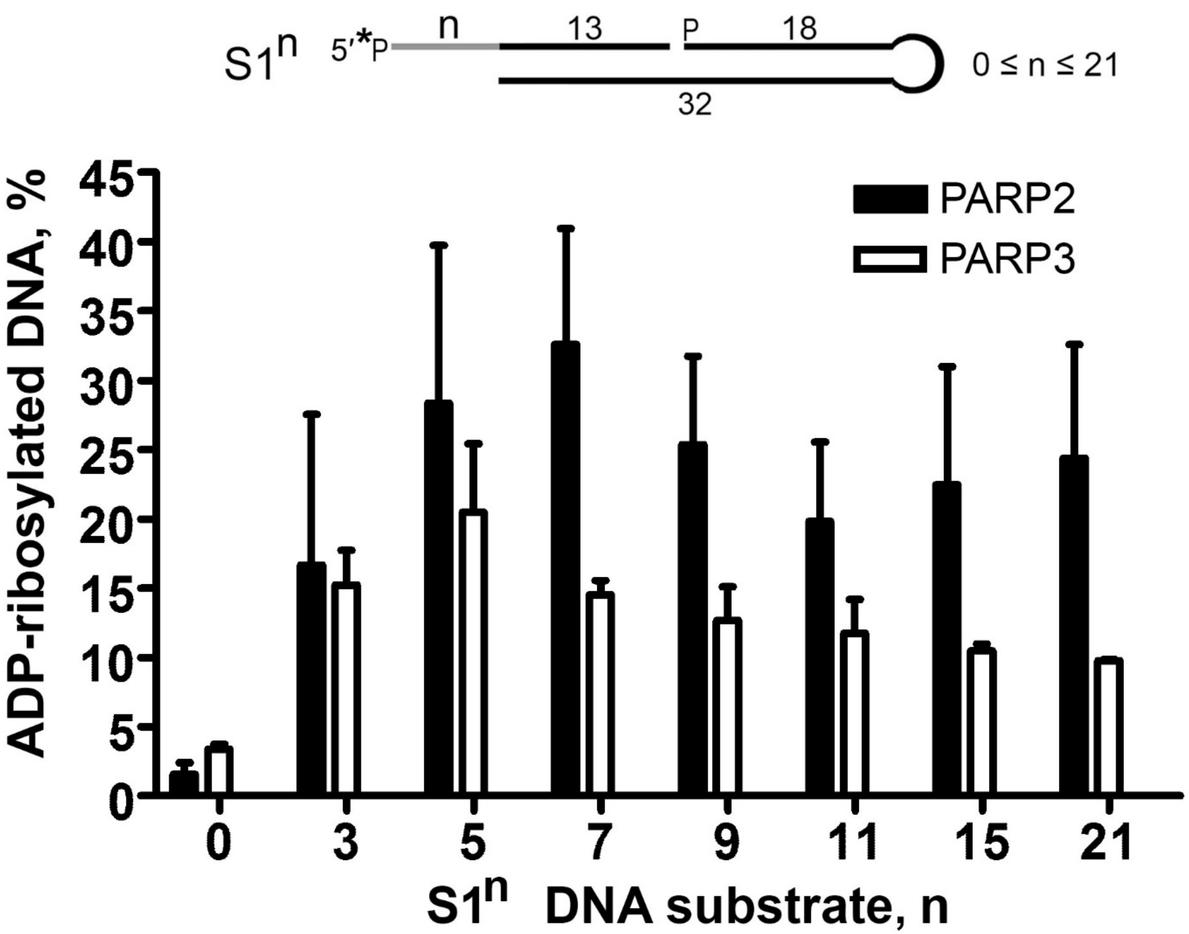

Figure S4. ADP-ribosylation of S1ⁿ DNA duplexes containing 5′-otherhangs by PARP2 and PARP3. [^{32}P]labelled S1^{n} DNA duplexes (20 nM) were combined with 50 nM PARP3 or PARP2 in the presence of 1 mM NAD^+ in ADPR buffer (20 mM HEPES-KOH pH 7.6, 50 mM KCl, 5 mM $MgCl_2$, 1 mM DTT and 100 µg/mL BSA). The mixture was incubated for 10 min (PARP3) or 30 min (PARP2) at 37°C. The data on PARP-catalysed formation of DNA ADP-ribosylation products are presented as mean ± SD from three independent experiments.
